# Prevalence of skin problems and leg ulceration in a sample of young injecting drug users

**DOI:** 10.1186/1477-7517-11-22

**Published:** 2014-08-13

**Authors:** Alison F Coull, Iain Atherton, Avril Taylor, Andrew E Watterson

**Affiliations:** 1 School of Nursing Midwifery and Health, University of Stirling, Stirling FK9 4LA, UK; 2 Faculty of Education, Health and Social Sciences, University of West of Scotland, Paisley PA1 2BE, UK; 3 Centre for Public Health and Population Health Research, University of Stirling, Stirling FK9 4LA, UK

**Keywords:** Leg ulceration, Skin, Prevalence, Drug injecting

## Abstract

**Background:**

Drug users suffer harm from the injecting process, and clinical services are reporting increasing numbers presenting with skin-related problems such as abscesses and leg ulcers. Skin breakdown can lead to long-term health problems and increased service costs and is often the first indication of serious systemic ill health. The extent of skin problems in injecting drug users has not previously been quantified empirically, and there is a dearth of robust topical literature. Where skin problems have been reported, this is often without clear definition and generic terms such as ‘soft tissue infection’ are used which lack specificity. The aim of this study was to identify the range and extent of skin problems including leg ulceration in a sample of injecting drug users. Definitions of skin problems were developed and applied to descriptions from drug users to improve rigour.

**Methods:**

Data were collected in needle exchanges and methadone clinics across Glasgow, Scotland, from both current and former drug injectors using face-to-face interviews.

**Results:**

Two hundred participants were recruited, of which 74% (*n* = 148) were males and 26% (*n* = 52) were females. The age range was 21–44 years (mean 35 years). Just under two thirds (64%, *n* = 127) were currently injecting or had injected within the last 6 months, and 36% (*n* = 73) had previously injected and had not injected for more than 6 months.

Sixty per cent (*n* = 120) of the sample had experienced a skin problem, and the majority reported more than one problem. Most common were abscesses, lumps, track marks and leg ulcers. Fifteen per cent (*n* = 30) of all participants reported having had a leg ulcer.

**Conclusions:**

This is an original empirical study which demonstrated unique findings of a high prevalence of skin disease (60%) and surprisingly high rates of leg ulceration (15%). Skin disease in injecting drug users is clearly widespread. Leg ulceration in particular is a chronic recurring condition that is costly to treat and has long-term implications for drug users and services caring for current or former injectors long after illicit drug use has ceased.

## Background

The skin is the largest and heaviest organ of the body and provides protection against pathogens and aqueous, chemical and mechanical assault, and has a role in metabolism, thermoregulation, sensation and communication [1]. By injecting with a needle, the body’s main protective barrier, the skin, is breached potentially causing harm. Illicit drug injectors repeatedly breach this barrier and may use contaminated drugs and unsterile equipment causing injecting injuries. A plethora of risk factors may contribute to harm and subsequent skin problems, such as injecting technique, the injecting mixture, quantity of acid for dissolving, choice of injecting equipment and paraphernalia, selection of injecting site, longevity of injecting habits as well as underlying health and social issues [2,3]. Various skin problems in drug users have been reported such as abscesses, ulcers, track marks and soft tissue infections [2,4-6]; however, the published papers tend not to adequately define or explain these skin complications [7,8]. Reported skin problems are at best ill defined but more commonly grouped together as soft tissue infections without specifying what type of lesions or describing the injury clearly such that consistent clinical diagnosis or comparisons between studies can be made [9-12]. Of concern is the grouping together of open wounds as ‘infections’ without identifying key features of skin infection such as pain, redness, pus and cellulitis as this may lead to inappropriate diagnosis and unnecessary prescribing of antibiotics [3]. Similarly, positive bacterial cultures from skin lesions do not always indicate clinical infection as most open wounds will contain bacteria but are not necessarily infected [13]. Differentiating between types of wounds is therefore important for treatment especially where drug users tend to attend and be treated in drug services rather than specialist wound centres.

The extent, or prevalence, of skin problems in the drug-using population was previously unknown. A literature search conducted in 2012 revealed no relevant empirical studies, so there is a dearth of evidence about not only the magnitude of the problem but also the short-and long-term implications of skin breakdown for injecting drug users, although skin disease is a known and frequent cause of morbidity in the homeless population, many of whom are drug users [14,15]. Health care professionals are increasingly encountering injection-induced wounds [16,6,19,5] such as infections, abscesses and leg ulcers in their practice, but much of the published literature focused on skin and wound care specifically is limited to single case reports (for example [16,20]). Skin breakdown, whilst serious in itself, is often the first indication of a serious systemic disease process such as septicaemia, anthrax or necrotizing fasciitis [21-25], so understanding the implications of injecting on the skin is important [12,26].

Although no empirical quantitative data exists, case studies and anecdotal reports also indicate that phlebitis, thrombosis and clots in the venous system occur frequently in drug users as a sequelae to injecting [27,19,32], and lasting damage to skin, such as chronic ulceration from venous insufficiency, may result. Many users report periods of hospitalisation due to deep vein thrombosis which damages vein valves [33,31]. Venous disease, and post-thrombotic syndrome, may be displayed in relatively young individuals who have been, or are, drug injectors [28]. Chronic venous disease leads, at its end stage, to ulceration of the skin, which, in the non-drug-using population, tends to become long lasting and recurs [34]. The progression of venous disease in drug users is unknown, but it is likely to be similarly persistent and costly [35].

Leg ulceration is arguably the most costly of injecting injuries to the skin with long-term implications for treatment and medical care. Around 1% of the general population will suffer from leg ulceration at some point in their lives [36]. Leg ulceration can also have an impact on quality of life with sufferers citing pain, odour and mobility problems. Injecting drug users are seen increasingly at leg ulcer clinics, but it is unknown how significant the problem in this population is or what the long-term sequelae may be [2,35].

The increasing numbers of case studies published [37,38], anecdotal reports and related studies [39,5,19,18] would indicate that the presence of skin disease in injectors is becoming more prevalent or apparent which would suggest the need for further empirical study.

Clinical observations amongst the drug-using population in Glasgow have demonstrated a prevalence of skin problems and leg ulceration [2], but no one appears to have previously examined and quantified the extent of the problem.

The aim of this study, therefore, was to identify the prevalence of skin problems and of leg ulceration, within a convenience sample of intravenous drugs users in Glasgow.

## Methods

A survey was undertaken and data were collected using structured face-to-face interviews utilising a specially designed questionnaire. The questionnaire was developed based on knowledge of injecting habits drawn from literature and anecdotal experience and aimed to identify likely risk and causal factors. Participants were asked questions about their injecting habits and complications related to skin that they had experienced during their injecting careers. As no published definitions of injecting wounds could be found that were comparable, definitions of known skin complications arising from injecting were developed and refined to ensure rigour based on existing literature and clinical expertise [40,2,41]. Utilising the same researcher and the same set of definitions ensured maximum rigour and accuracy in the reporting of the skin problems, within the limitations of self-report and recall. Participants were asked for clarification of the meaning of words they used to describe injecting injuries such as ‘abscess’ or ‘acid burn’, in order that the same definitions were applied to the same type of description.

The definitions were matched to descriptions from the participant to ensure that the same definition was applied to each case (Table 1). The position of lower extremity wounds was ascertained to avoid confusion between the reporting of foot wounds and the reporting of leg ulcers. Previous work has recognised the importance of clarity of position of wounds especially when the term ‘ulceration’ is used which can be misunderstood [42]. Participants who experienced skin problems were asked further questions about the type of skin problem they had. These were defined and explored with each participant to ensure that a standard definition (see Table 1) was adhered to.

**Table 1:** Definitions of skin problems

**Skin problem**	**Definition**
Leg ulcer	A break in the skin between the knee and the ankle that remains unhealed for 4 weeks or more (SIGN, 1998)
Lumps	Hard swellings without broken skin, not red or hot or particularly painful
Track marks	Scratch marks, raised red veins, raised hardened veins
Abscesses	Raised red hot painful lumps, with or without obvious pus/broken skin—possibly required lancing/surgery or have spontaneously burst
Acid burns	Painful, blistered or broken skin directly attributed to use of acid
Broken skin (heals within 4 weeks)	Injecting injury that has caused a break in the skin, wounds or scabs that have healed in less than 4 weeks
Chronic wounds	Any break in the skin (not a leg ulcer) that has been present 4 weeks or more
Rashes	Multiple red or pink spots, raised or flat, that last longer than the short period following injection

A number of additional questions were included (related to injecting habits and risk factors); however, only the prevalence data are reported in this paper.

### Sample

Drug use has its own subcultures and users often educate peers within their own geographical areas, and actual injecting practices can vary from suburb to suburb. A wide geographic sample even within one city, therefore, was important to ensure variations due to locality were captured. Participants were recruited from eight different venues (needle exchanges and methadone clinics) in the north, south, east and west of Glasgow. These venues were selected as they were where the highest numbers of injecting drug users were known to engage with services in each direction of the city and where a private room was available for interviewing. A convenience sample was selected as this is a challenging population to investigate because many drug users are transiently accommodated, frequently incarcerated and engaged in chaotic lifestyles, which means arranging appointments for interview or follow-up can be difficult or impossible, and a sampling frame could not therefore be used [43,44,6]. The approach to recruitment was similar to that of other local studies which had successfully overcome many of the traditional problems of recruitment of drug users [45].

### Inclusion/exclusion criteria

In Scotland alone, it is thought that the number of individuals with problem drug use is nearly 60,000. Glasgow has the highest rate of problematic drug use in Scotland, with prevalence rates remaining consistent in the last 5 years and thought to be around 13,900 individuals. The majority of drug users are aged between 15 and 64 years, with the greatest proportion in the 35–64-year age group [46]. The prevalence of injecting drug use is not currently known, although older studies have been published [47].

The research aimed to capture people like these described, whilst avoiding the challenges of working with children. Participants over the age of 44 years were excluded to attempt to reduce the potential results created by the impact of age-related disease on ulcer development. Leg ulcer studies of a wider population will tend to include from age 50 years of age or over [42], so 44 years was considered to be a safe margin.

The sample included individuals with a current or previous history of injecting drug use, aged 16 to 44 years, who could understand and speak English. Participants were excluded if they did not meet the inclusion criteria or did not appear to be able to either understand what they are consenting to take part in or answer questions, including being visibly under the influence of drugs or alcohol. Staff at the recruitment venues who were often familiar with individual participants also helped identify and exclude those who were not fit to consent at that time [48].

### Recruitment

Posters and leaflets describing the study were displayed in each venue for at least 2 weeks prior to recruitment commencing. During recruitment, individuals were approached by the researcher as they entered each venue and asked if they would be interested in taking part in a research study and answering some questions about injecting and complications. Informed consent was obtained and the interviews lasted an average of 20 min.

A pilot study was undertaken earlier to test recruitment, data collections tools and analysis which informed the method. Ethical and governance approval was sought and obtained from NHS Greater Glasgow and Clyde to undertake the study.

The study was conducted over a period of 3 months at the end of 2008.

### Data collection

Interviews were conducted across the eight venues in the north, south, east and west of the city. Participants provided initials and date of birth, together with the first few letters of their postcode—this allowed any duplicate interviews to be identified.

All interviews were conducted by one researcher, and participants were given an honorarium of a £2 shopping voucher as an acknowledgement of their time taken to participate.

A total of 204 interviews were conducted. On three occasions, participants who the researcher thought had been interviewed before were adamant they had not previously been interviewed. The researcher felt obliged to conduct a second interview. When initials and dates of birth were checked, the three interviewees were found to be duplicates. The participants knew of the incentive/honorarium and may have wished to collect another £2 voucher. The data from these second three interviews were removed from the database. A fourth interview was abandoned midway, and the incomplete data from the fourth interview were also removed and not included in the analysis. The analysis was conducted on the remaining 200 interviews.

### Data analysis

All data were entered into SPSS version 15.0 and checked independently for accuracy. All identifiers were removed after checking. Data were analysed using descriptive and inferential statistics. The confidence interval for all tests was 95%. The data were reviewed for missing values and there were none.

## Results

### Sample

As can be seen from Table 2, participants were predominantly male (74%, *n* = 148) with 26% (*n* = 52) females. The age range was 21–44 years (mean 35 years). Just under two thirds (64%, *n* = 127) were currently injecting or had injected within the last 6 months, and 36% (*n* = 73) had last injected more than 6 months prior to the interview.

**Table 2:** Summary of results

		**Injected within the last 6 months ( *n *)**	**Percentage**	**Previously injected but not in the last 6 months ( *n *)**	**Percentage**	**Total ( *n *)**	**Percentage**
All		127	64	73	36	200	100
Gender	Male	97	76	51	71	148	74
	Female	31	24	21	29	52	26
						200	Total
Age group (years)	20–24	7	5.5	4	5	11	5.5
	25–29	23	18	6	8	29	14.5
	30–34	39	31	11	15	50	25
	35–39	37	29	28	38	65	32.5
	40–44	21	16.5	24	33	45	22.5
						200	Total
Age when started injecting (years)	Under 16	21	16	14	19	35	17.5
	16–19	29	23	28	38	57	28.5
	20–24	38	30	17	23	55	27.5
	25–29	25	20	7	10	32	16
	30–34	10	8	7	10	17	8.5
	35–39	4	3	0	0	4	2
						200	Total
Length of injecting career (years)							
	Less than 1	7	5.5	12	16	19	9.5
	1–5	32	25	15	21	47	23.5
	6–10	35	27.5	14	19	49	24.5
	11–20	35	28	24	33	59	29.5
	Over 20	18	14	8	11	26	13
						200	Total
Individuals reporting a skin problem		80	63	40	55	120	60
Type of skin problem	Leg ulcer	17	13	13	18	30	15
	Lumps	40	31	18	25	58	29
	Track marks	40	31	16	22	56	28
	Abscesses	58	46	32	44	90	45
	Acid burns	21	17	8	11	29	15
	Broken skin	15	12	10	14	25	13
	Chronic wound	17	13	11	15	28	14
	Rashes	2	2	1	1	3	2
	Other skin problems	6	5	5	7	11	6

Some individuals reported more than one skin problem so therefore percentages may add up to more than 100%. Percentages rounded up.

Of the current injectors, 20% were long-term injectors (*n* = 26), who had been injecting for 20 years or more.

In response to the question ‘Have you ever had a skin problem?’, 60% (*n* = 120) said yes and 40% said no (*n* = 80). The majority of these were male (72%, *n* = 86; females 28%, *n* = 34).

The majority of the current (63%, *n* = 80) and over half of the former (55%, *n* = 40) injectors reported a problem with the skin. Almost all of the long-term injectors (>20 years) reported a problem with their skin (85%, *n* = 22).

**Figure 1:**
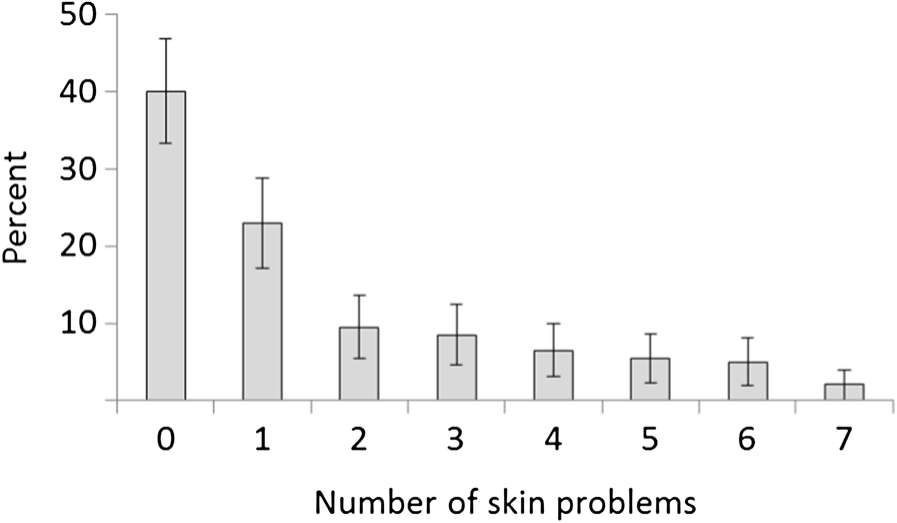
Number of different types of skin problems experienced.

A Mann-Whitney *U* test comparing groups of those that had injected for less than a year, 1–5 years, 6–10 years, 11–20 years and over 20 years showed that those who injected for longer experienced more skin problems (*U* = 2,896, *z* = −4.89, *p* = ≤0.001), indicating that the longer a person injects, the more likely they are to have a skin problem.Although 60 participants had never had skin problems, 74 (62%) of the participants reporting skin problems experienced more than one type of problem (Figure 1).

The majority of those who reported skin problems complained of abscesses (75%, *n* = 90), followed by lumps (48%, *n* =58) and track marks (47%, *n* =56), followed by leg ulcers (25%, *n* = 30) acid burns (24%, *n* = 29) and chronic wounds other than leg ulcers (23%). Eight per cent complained of other skin problems, and these were scarring (*n* =3), bruising (*n* =2), varicose veins (*n* =2), phlebitis (*n* =1), cellulitis (*n* =1), phlebitis (*n* = 1), necrotising fasciitis (*n* =1), haematoma (*n* =1) and thin skin (*n* = 1).

In response to the question ‘Have you ever had a leg ulcer?’, 25% (*n* =30) of those reporting skin problems said yes; of these, 47% (*n* =14) had active ulceration at the time of the interview. Therefore, the prevalence of active leg ulceration within the 200 participants was 7% (14/200) and lifetime prevalence was 15% (30/200). Eighteen per cent (*n* = 13/73) of the former injectors and a total of 13% (*n* = 17/127) of the current injectors had suffered leg ulceration.

A higher percentage of females had leg ulceration (17%, *n* = 9/52) than men (14%, *n* = 21/148). Of those with a leg ulcer, 6 ulcers had never healed, 12 ulcers had healed up and broken down/recurred again and 12 ulcers had healed up at the time of the research.

## Discussion

This study is the first empirical work that accurately defines and matches described skin problems in injecting users to specified terms. Drug users with skin problems are most likely to present to services such as needle exchanges and drug workers in the first instance, where staff have less expertise in the physical side of injecting complications, such as the identification of wound infection, and the management of acute and chronic wounds. It has therefore been difficult, until now, to empirically determine the nature and extent of skin problems because of the diverse presentation across the sector.

The clear, but simple, definition of skin problems is new in the field and ensures consistency and accuracy by researchers, within the scope of participants’ recall. All skin problems within this study were self-reported by participants. Reassuringly, a closely relevant study by Morrison et al. [49] compared answers from a self-report questionnaire about injecting-related harm to clinicians’ observational findings and found consistency between the two. Whilst the definitions were stringently applied to the data collection, some skin problems may have been forgotten, or exaggerated. However, the evidence suggests that recall is fairly accurate, even in drug users [50-52].

There was an advantage in gathering data face to face in that the researcher was able to clarify and determine the precise condition reported by the participants by probing further and seeking clarification on position as well as appearance. However, clinical examination was not undertaken; this may have been advantageous where there was a current skin problem but would not have assisted in identifying or clarifying past problems. However, the method had to be constructed to suit the population studied.

### Sample

The socio-demographic profile of Glaswegian drug injectors is known to be two-thirds male, typically unemployed and aged about 30 years, and mostly using heroin [53-55]. The sample in this study was similar in that the majority of participants were male with a comparable mean age.

There were no participants under the age of 21 years, and no one was excluded on the basis that they were too young to participate. This may indicate that injecting in this younger age group is low and so skin problems may be small or non-existent or that the services used for recruitment were not engaging with younger drug users and so this figure may not be indicative of skin problems in younger injectors.

### Skin problems

One hundred twenty participants reported skin problems, giving a prevalence of 60%. The majority complained of abscesses (75%), lumps (48%) and track marks (47%), followed by leg ulcers (25%), acid burns (24%) and chronic wounds other than leg ulcers (23%). Eight per cent complained of other skin problems over the duration of their injecting careers including bruising and varicose veins, phlebitis, cellulitis, haematoma, scarring and thin skin.

These findings indicate that a large proportion has experienced abscesses and soft tissue infection. This is unsurprising given that the injection process will not be a sterile procedure and the drugs injected are often cut and mixed with unsterile materials; nevertheless, the prevalence is extremely high.

Any injecting injury is frequently referred to as an abscess by both service users and staff. Similarly, within the medical literature, soft tissue/bacterial infection is the main injecting problem described [56]. Whilst there is no denying that infection is a common sequela to injecting illicit materials, this is a misrepresentation of the problem.

Traditionally, abscesses may be defined as ‘a localised collection of pus’ [57]. However, many papers commonly use the term abscess to describe any kind of red raised lump. This is confusing and possibly harmful. Many missed ‘hits’ (injections) also appear as red lumps and can be misnamed abscesses. The injection may miss the vein, or puncture the vein, allowing fluid to escape into the tissues. They tend not to be pus filled, hot, or with spreading cellulitis; however, they may take some time to resolve but are not infected and do not require antibiotics [58]. The abscesses may require treatment with antibiotics, but overuse or inappropriate use of antibiotics can lead to resistance and other unwanted side effects [13].

However, appropriate assessment of skin problems and injecting injuries is important as misdiagnosis can have serious implications. Skin breakdown may be the first sign of problematic drug use. Beeching and Crowcroft [59], in their overview of tetanus in injecting drug users (IDUs), warn clinicians of systemic effects of apparently trivial wound infections in IDUs when they present with collapse, sepsis or odd neurological symptoms which might otherwise be dismissed as direct results of drug intoxication. Seemingly innocuous lesions can also indicate far more serious systemic disease (e.g. anthrax, wound botulism, clostridia [60,4,61]), and skin problems must not be viewed in isolation and should be part of a systemic assessment of other signs and symptoms such as fever and flu-like illness. It is normal for the body to respond with a temporary inflammatory response to injury, but what is more difficult is detecting the difference between normal inflammation and an inflammatory process which may indicate something more serious and systemic occurring.

Existing guidance errs very much on the side of caution, which, although correct, demonstrates the difficulty in differentiating between lesions, and partly why definition is so important [62]. Clearer guidance is needed for both health professionals and drug users on skin problems.

### Leg ulceration

The findings revealed a high prevalence of leg ulceration in the sample population of young injecting drug users. Fifteen per cent of the sample population had experienced a chronic leg ulcer, defined as ‘a break in the skin between the knee and the ankle that was present four weeks or more’ [63], whilst 7% had an active ulcer at the time of interview. Comparatively, within the general population, it is known that 1% of the adult populations (within Western countries) are likely to have a chronic leg ulcer at some time [64]. Later studies have agreed with this figure [65,66], and therefore, the prevalence of leg ulceration within an injecting population is worryingly high. This figure, of 15% prevalence, verifies the anecdotal claims from community nursing practitioners who are seeing rising numbers of young people with leg ulceration in their clinical practice but have little empirical evidence to support their assertion [67].

This finding is of concern as once ulceration occurs, the long-term impact on health services may become onerous. The ulcerations in the study were occurring in younger people than those who traditionally form the 1% of the population that experience leg ulceration. Generally, this is a disease of old age and prevalence is known to increase with age [68]. Whilst considered a disease of the elderly population, it is not rare to develop ulceration before middle age. Work by Mackenzie et al. [69] identified that in a sample of 118 patients with leg ulceration, 46% had had developed ulceration before the age of 50 years and were more likely to be male and obese and to have a history of DVT and/or long bone fracture. In mainstream studies of leg ulceration, it is possible that injecting drug use is not considered as a risk factor, especially if illicit use is hidden for example in femoral injectors [70].

However, if younger people are increasingly experiencing leg ulceration, then the impact on health services will be pronounced as recurrence occurs and as sufferers age. Leg ulceration is end-stage venous disease [34] and a chronic recurrent condition which is costly, not just in terms of treatment but long-term prevention of recurrence and also in terms of human suffering [71]. The ulcers can be painful, malodorous and debilitating, badly impacting on quality of life and costly to treat [72-74]. The impact on younger people may arguably be more severe, preventing normal activities and possibly even employment, and the ulceration may affect lives long after drug use has ceased [75].

Treatment for venous ulcers in a more elderly population consists of regular and frequent intervention, usually with specialised compression therapy requiring expert nursing staff and considerable time to achieve healing. Generally, compression bandaging is followed with long-term compression hosiery. Frequent appointments for treatment and long-term follow-up can be challenging to achieve amongst a drug-using population with chaotic lives who often have unstable accommodation arrangements, and there are no specific guidelines to help practitioners manage this particular population. Of course, the life expectancy of an injecting drug user may be reduced, and therefore, the figures for long-term injectors and older injectors in this study need to be interpreted with caution due to reduced life span and bias as a result of early death [76].

Within the general population, women tend to develop leg ulceration more commonly than men [77,75], and this increased prevalence is borne out within this study. It has been suggested, although not conclusively, that this is due to hormonal differences or longevity [78]. In drug injectors, it has been suggested that women’s veins become damaged and incompetent quicker than men as they tend to be smaller and thinner walled [79]. It may also be that women may tend to be more determined to hide the visual evidence of injecting and opt to inject in the groin which impacts on disease processes in the lower leg [6].

Femoral injecting may increase the risk of deep vein thrombosis due to repeated puncture, scarring and narrowing of the femoral vein [32], and one of the complications may be distal venous disease, including leg ulceration. In Glasgow, femoral injecting is relatively common and the link between using this site and the high rate of leg ulceration is worth exploring in the future.

## Conclusion

This original paper reports on a unique empirical study to determine the prevalence of skin problems and leg ulceration in a sample of intravenous drug users. This is the first paper that the authors are aware of that sets out to carefully define skin lesions caused by injecting and quantify these within a sample.

This study demonstrates that skin problems are a significant, widespread issue for young injecting drug users. It has revealed a high prevalence rate of leg ulceration that is alarming. As leg ulceration is considered an end-stage venous disease, it is reasonable to expect that as intravenous drug users age, they will create a significant proportion of patients requiring lower limb care. We know leg ulcer care is costly [73], and the public health implications of these chronic open wounds, which can create dependent individuals with pain and impaired mobility, are significant. Further studies are required to examine the risk factors and the role of specific harm reduction activities such as dispensing of alcohol wipes for skin cleansing and distribution and use of sterile equipment, together with a closer examination of injecting techniques such as licking of needles, flushing and use of particular sites directly linked to skin problems, in more depth.

Harm reduction initiatives have recently focused on reducing the spread of blood-borne viruses but have failed to address the direct harm as a consequence of injecting. Raising awareness of wounds and skin breakdown in injecting drug users and health professionals may help with earlier detection and intervention to combat more serious disease or the development of more life-threatening complications.

## Competing interests

The authors declare that they have no competing interests.

## Authors’ contributions

AFC conceived and conducted the study, gathered the data, undertook the analysis and drafted the manuscript. IA undertook some statistical analysis. IA, AT and AEW supervised the study and revised the manuscript. All authors have approved the final draft of the manuscript.
